# From Pseudotumor Cerebri to Neurobrucellosis: A Journey With Several Lessons

**DOI:** 10.7759/cureus.57496

**Published:** 2024-04-03

**Authors:** Moammar AL Aamri, Vivek Mathew, Shahid Iqbal, Suad AL Mukhaini

**Affiliations:** 1 Internal Medicine, Sultan Qaboos Hospital, Salalah, OMN; 2 Neurology, Sultan Qaboos Hospital, Salalah, OMN; 3 Infectious Disease, Ministery of Health, Salalah, OMN

**Keywords:** meningitis, csf pleocytosis, brucella, neurobrucellosis, pseudotumor cerebri, idiopathic intracranial hypertension

## Abstract

We present a case admitted for evaluation of suspected idiopathic intracranial hypertension (IIH) with an unusual but important departure from the expected algorithm. A 31-year-old lady came with a two-week duration of a mild headache and one-week duration of double vision with no previously documented fever or any comorbidities. Clinically, she had papilledema and bilateral abducens palsy with no signs of meningeal irritation. MRI brain radiology was consistent with IIH. Her CSF study showed pleocytosis with elevated protein levels and normal glucose. Serology was positive for *Brucella melitensis* at low titers but CSF culture grew *Brucella melitensis*, confirming the diagnosis of neurobrucellosis. Her headache and abducens palsy improved over the first two weeks, and the papilledema resolved over two months with antibiotics. This clinical mimic is important for physicians (including neurophysicians) and Infectious Disease specialists. The radiological mimic comes from chinked (small) ventricles, unlike most meningeal diseases which can present with papilledema and abducens palsy including tuberculosis, cryptococcosis, and leptomeningeal carcinomatosis. A CSF study is mandatory in the workup of IIH despite massive improvements in imaging.

## Introduction

Idiopathic intracranial hypertension (IIH), previously known as benign intracranial hypertension or pseudotumor cerebri, is a disorder that is most commonly observed in young obese or overweight women. The magnetic resonance imaging (MRI) findings of IIH have been well described and accepted [1]. Our Neurology clinic manages 100-120 patients every week, including 2-8 patients with IIH, of whom 0-2 are newly diagnosed. In this department, the modified Dandy criteria is followed as an algorithm for a definitive diagnosis, and a follow-up plan is made according to vision parameters, particularly in the field [2]. The MRI findings of IIH are sensitive but not specific [3]. These symptoms can be found in many overweight young women, even those without headaches or papilledema. However, when a patient with headache and papilledema (with or without abducens paresis) presented with MRI features of IIH, and for whom other causes of increased intracranial pressure had been ruled out, lumbar puncture was chosen as the next step. The main purpose of the cerebrospinal fluid (CSF) study was not manometry; rather, it aimed to demonstrate normal CSF content. The clinical question raised during the evaluation of the patient presented here was how to approach an abnormal CSF report in a patient with otherwise fairly typical IIH. This brought us to a diagnosis of another reasonably common condition in the Dhofar Governate of the Sultanate of Oman. Brucellosis is a common zoonotic infection worldwide. It is particularly prevalent in the Middle East [4]. Close contact with domestic animals and ingestion of raw dairy products and meat from livestock are the major routes of transmission. The Dhofar region in southern Oman has a cooler climate and monsoon season (Khareif). As this region is rich in livestock, Brucellosis condition is endemic in this area [5].

Brucellosis involves multiple systems and exhibits a wide range of clinical features [6]. The symptoms and signs depend on the stage of the disease and the organs or systems involved. Central nervous system (CNS) complications are uncommon. However, they can be serious, and this diagnosis should be suspected in patients from endemic regions who present with clinically atypical neurological symptoms and signs [7]. Pseudotumor cerebri as a presentation of neurobrucellosis is extremely rare; in Oman, it has only been reported once in the paediatric age group [8]. Herein, we report an adult case of neurobrucellosis in Oman, who presented with symptoms of IIH.

## Case presentation

A 31-year-old woman was admitted to our Neurology outpatient department (OPD) following a referral to our ophthalmology department for the evaluation of papilledema with bilateral abducens palsy. She reported double vision lasting for one week. On leading questions, she mentioned a mild headache starting approximately two weeks prior. She had no history of fever, ear discharge, eye redness, or sensorial alteration. Further, she had no recent or past chronic illnesses and was not taking any medication. 

The patient was seated comfortably in the OPD. Subsequent examination revealed severe bilateral papilledema in the form of a congested swollen disc with obliterated disc margins. She further had left-sided bilateral abducens nerve palsy. Other cranial nerves were normal, and she had normal coordination and gait, with no lateralizing long tract signs. No meningeal irritation was observed. 

**Figure 1:**
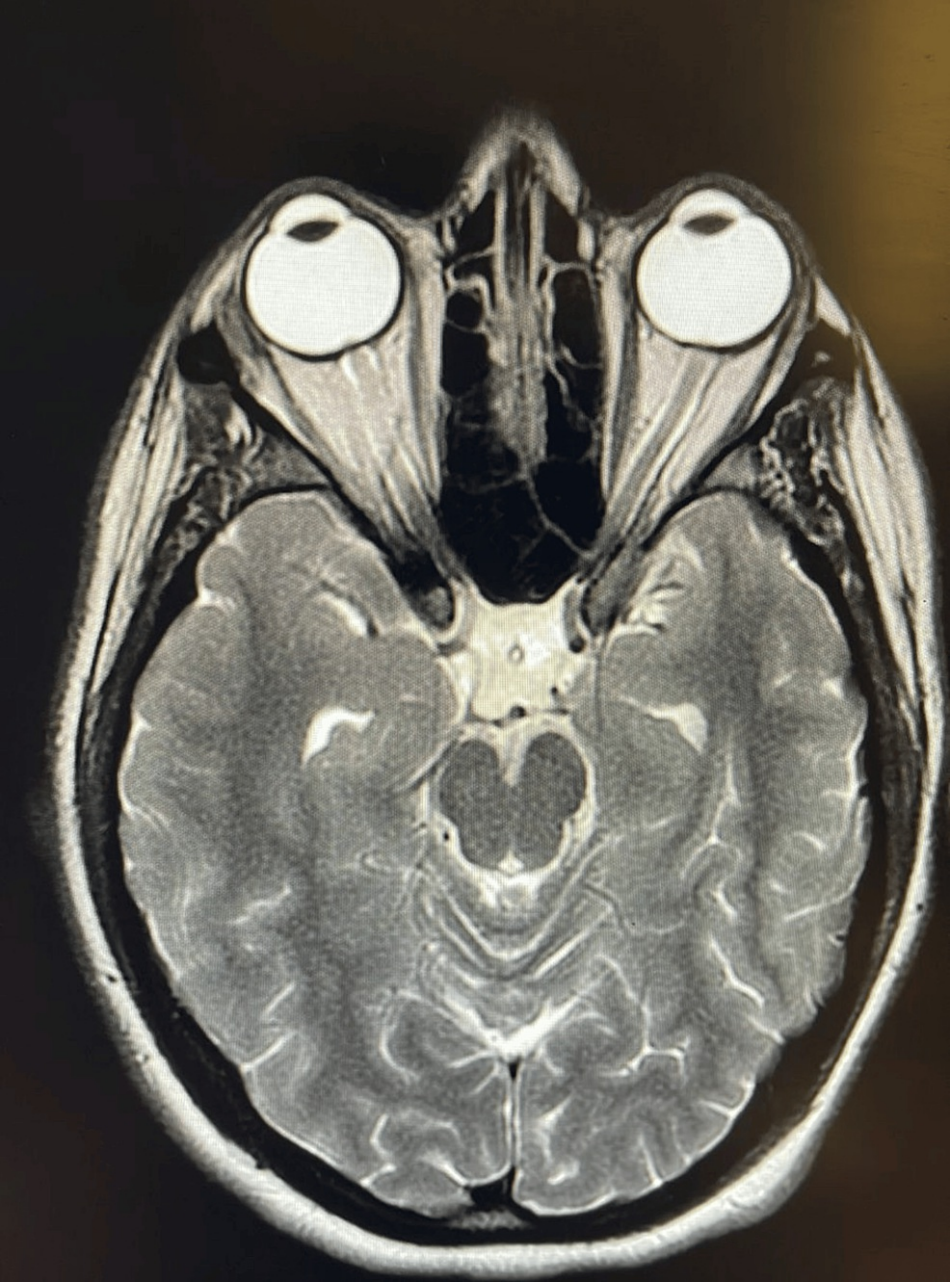
Axial T2 weighted MRI showing a distended optic nerve sheath and flattening of the globe at the disc

**Figure 2:**
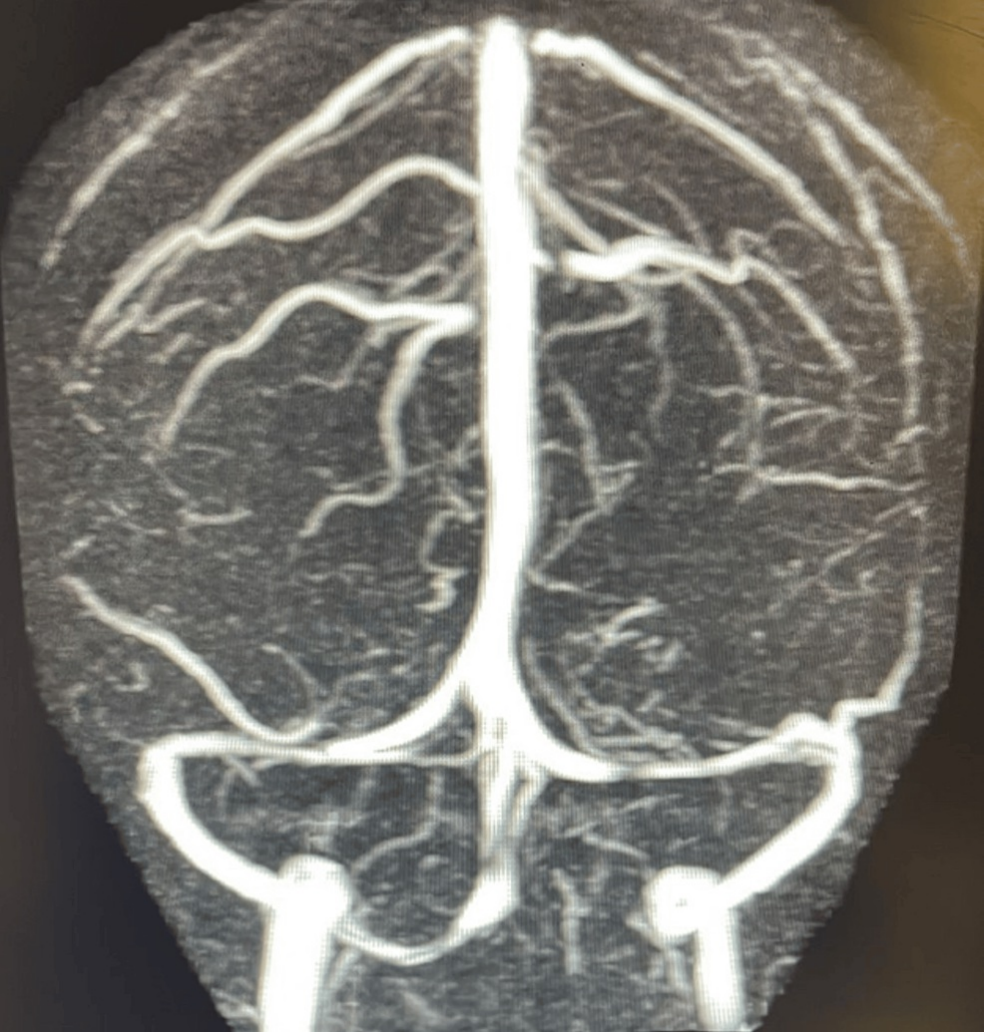
MR venogram showing narrowing of the bilateral transverse sinus and no signs of cerebral venous sinus thrombosis

**Figure 3:**
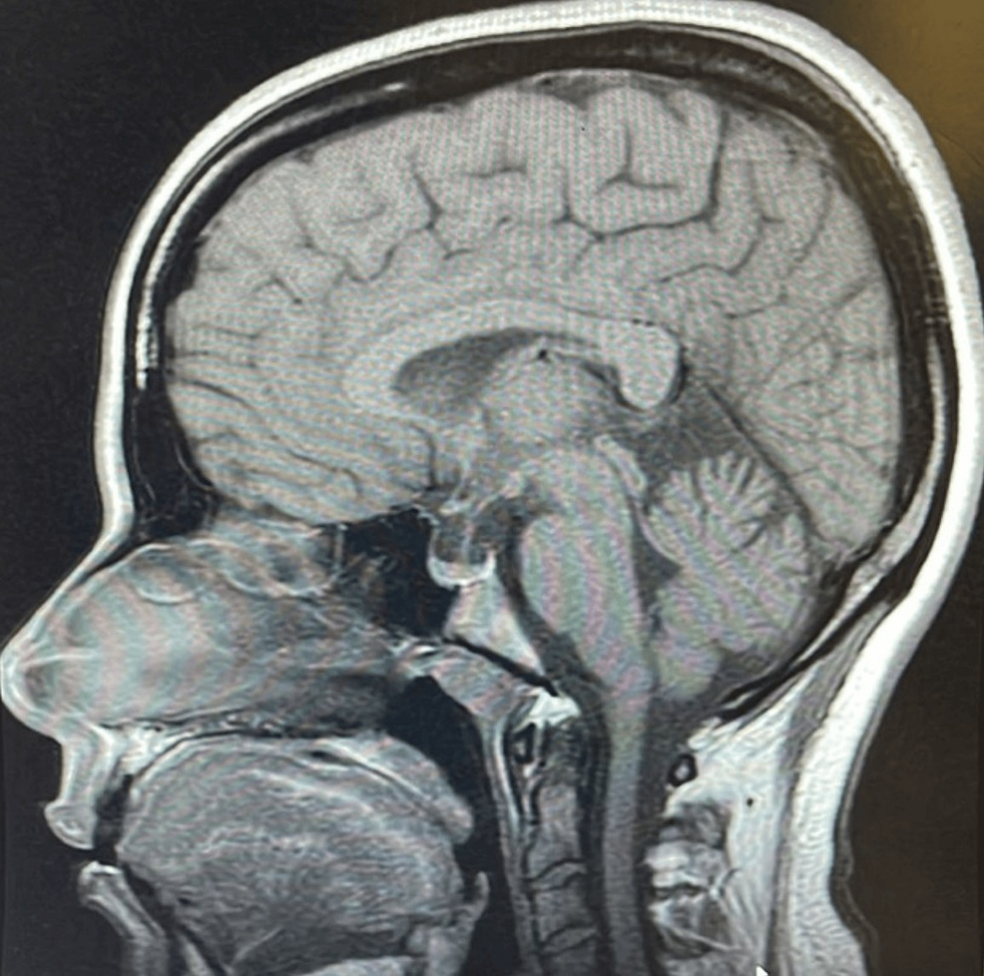
Sagittal T1 weighted MRI showing a partially empty sella

The ophthalmology department had already ordered an MRI of the brain, which had been performed the previous evening. MRI (Figures 1-3) revealed a distended optic nerve sheath, flattening of the globe at the disc, and a partially empty sella, with narrowing of the bilateral transverse sinus. No signs of cerebral venous sinus thrombosis were observed. The Radiologist suggested a diagnosis of IIH for clinical and fundoscopic correlations. The patient was started on escalating doses of acetazolamide and advised to be admitted for lumbar puncture and CSF studies. Blood investigations revealed normal complete blood counts and normal liver and renal functions. The admitting diagnosis was possible IIH.

Lumbar puncture showed a high CSF opening pressure (>50 cm of CSF/water) in the lateral decubitus position. The results of CSF analysis were as follows: glucose 2 mmol/L, protein 117.60 mg/dl, white blood cells 188/mm^3^ (88% lymphocytes and 12% neutrophils), and red blood cells 5/mm^3^. Her serum random blood sugar was 6.9 mmol/L.

At this point, the symptoms of this young woman, including increased intracranial pressure and abnormal CSF findings, were more suggestive of infection than malignancy. She was therefore started on empirical antibiotics to treat bacterial meningitis and atypical organisms while awaiting the results of cultures and other tests. Injections of ceftriaxone 2g twice daily and ampicillin 2 g every 4 h, along with doxycycline tablets 100 mg twice daily and rifampicin capsules 600 mg once daily were initiated. The CSF was sent for bacterial culture, PCR, and tests for* Mycobacterium tuberculosis*, viral panel, and cryptococcal antigen. Serum was sent for blood culture, *Brucella* serology, HIV testing, and Treponema Pallidum Hemagglutination Assay (TPHA).

The Brucella agglutination test (Rose Bengal) was positive, with serology showing *Brucella abortus* 2ME of 1:320 and *Brucella melitensis* 2ME of 1:320. The CSF polymerase chain reaction (PCR) viral panel returned negative results at the Central Public Health Laboratory (CPHL). They tested for Adenovirus, HSV-1 and 2, VZV, Enterovirus, Parechovirus, Herpes 6 and 7, parvovirus, CMV, and EBV. On the seventh day of admission, the microbiology laboratory found growth of *Brucella melitensis* in the CSF culture leading to a final diagnosis of neurobrucellosis caused by *Bacillus melitensis*. She was discharged on the tenth day of admission to the local health center to complete a total of 4 weeks of injectable ceftriaxone 2 g twice daily, 12 weeks of oral rifampicin 600 mg once daily, and doxycycline 100 mg twice daily. Acetazolamide (500 mg, three times daily) was also administered. 

Follow-up was performed at the Neurology and Ophthalmology OPD at 4 and 10 weeks. The headache and double vision had resolved by the first visit, while papilledema had resolved completely by the second visit. The dose of acetazolamide was reduced to 250 mg three times and then twice daily to be tapered off completely by the next visit.

## Discussion

Herein, we report the case of a young woman who presented to the ophthalmology department due visual problems lasting one week, who was found to have bilateral abducens paresis and papilledema. Notably, the patient also had a history of mild headache one week prior to the onset of vision disturbances. These symptoms represent a classical presentation of raised intracranial pressure with no lateralizing or localizing deficits other than abducens paresis, which is considered a “false localizing sign.” This is the starting point of the algorithm for pseudotumor cerebri, originally discussed by Dandy in 1937 [9], and later modified by Smith in 1985 [10]. The name itself was initially changed to benign intracranial hypertension, and later to idiopathic intracranial hypertension because the unfortunate outcome of vision loss is not benign. 

The differential diagnoses for increased intracranial pressure without localizing signs are numerous. In order to allow diagnosis, our patient underwent brain MRI which showed features consistent with increased intracranial pressure with normal brain parenchyma, no extraparenchymal collections, no thrombosis in the venous sinuses, and no ventricular enlargement. With these radiological findings, minimal headache, and no documented temperature, it may have been tempting to maintain the patient on acetazolamide and perform follow-up, particularly if the patient or family is reluctant to undergo lumbar puncture. In this case, the patient and family agreed to undergo lumbar puncture, and the CSF findings suggested a meningeal process with lymphocytic pleocytosis, elevated protein, and low glucose levels. 

Due to the presence of minimal headache and normal-to-small ventricles, tuberculosis, cryptococcosis, and malignancy were considered unlikely in this case. Thus, we reviewed the literature on meningeal infections that mimic pseudotumor cerebri, with the results suggesting brucellosis and some viral causes [8,11-14]. Brucella serology was positive, but in our area, brucellosis is endemic, so this was not sufficient to establish the diagnosis. We therefore requested for CPHL to be assessed from an extended viral PCR panel, which returned negative results. Subsequent CSF culture revealed *Brucella melitensis*, thus confirming the diagnosis of neurobrucellosis. 

Brucellosis mimics many other conditions in terms of clinical manifestations. Meningitis and spondylodiscitis are well-recognized symptoms. In fact, due to the highly variable definitions used across the literature, disease at almost every location of the neuraxis has been attributed to brucellosis, with features ranging from psychosis, cerebral tumors, and aneurysms to Parkinsonism, CNS demyelination, and amyotrophic lateral sclerosis [15-20]. The present patients showed meningitis on CSF examination, but the clinical and radiological presentation only showed raised intracranial pressure and pseudotumor cerebri.

## Conclusions

In the case presented here, the diagnosis was confirmed by CSF culture, and we documented how neurobrucellosis can mimic IIH clinically and radiologically. Recent onset of headache and papilledema (with bilateral abducens palsy) were the only noted clinical presentations. Radiologically, IIH is associated with normal or chinked (small) ventricles, in addition to the imaging features of increased intracranial pressure. It is not yet clear why neurobrucellosis is associated with “chinked ventricles.”

Our experience, in this case, strengthens one component of the modified Dandy's criteria, namely the clinical algorithm for the management of a patient with suspected IIH. This case suggests that a CSF study is needed to exclude pleocytosis, in addition to documenting raised pressure. In other words, during the workup of a patient with suspected IIH, if CSF pleocytosis is encountered, neurobrucellosis needs to be considered.
